# Liquid Biopsy in Oligometastatic Prostate Cancer—A Biologist's Point of View

**DOI:** 10.3389/fonc.2019.00775

**Published:** 2019-08-14

**Authors:** Ewelina Stelcer, Marek Konkol, Aleksandra Głȩboka, Wiktoria Maria Suchorska

**Affiliations:** ^1^Radiobiology Laboratory, Greater Poland Cancer Centre, Poznan, Poland; ^2^Department of Electroradiology, Poznan University of Medical Sciences, Poznan, Poland; ^3^Department of Histology and Embryology, Poznan University of Medical Sciences, Poznan, Poland; ^4^Radiation Oncology Department, Greater Poland Cancer Centre, Poznan, Poland; ^5^Pharmacy, Greater Poland Cancer Centre, Poznan, Poland

**Keywords:** oligometastasis, prostate cancer, circulating tumor cell (CTC), microRNA, long-non coding RNAs, liquid biopsy

## Abstract

Prostate cancer (PCa) is the main cause of cancer-related mortality in males and the diagnosis, treatment, and care of these patients places a great burden on healthcare systems globally. Clinically, PCa is highly heterogeneous, ranging from indolent tumors to highly aggressive disease. In many cases treatment—generally either radiotherapy (RT) or surgery—can be curative. Several key genetic and demographic factors such as age, family history, genetic susceptibility, and race are associated with a high incidence of PCa. While our understanding of PCa, which is mainly based on the tools of molecular biology—has improved dramatically in recent years, efforts to better understand this complex disease have led to the identification of a new type of PCa–oligometastatic PCa. Oligometastatic disease should be considered an individual, heterogeneous entity with distinct metastatic phenotypes and, consequently, wide prognostic variability. In general, patients with oligometastatic disease typically present less biologically aggressive tumors whose metastatic potential is more limited and which are slow-growing. These patients are good candidates for more aggressive treatment approaches. The main aim of the presented review was to evaluate the utility of liquid biopsy for diagnostic purposes in PCa and for use in monitoring disease progression and treatment response, particularly in patients with oligometastatic PCa. Liquid biopsies offer a rapid, non-invasive approach whose use t is expected to play an important role in routine clinical practice to benefit patients. However, more research is needed to resolve the many existing discrepancies with regard to the definition and isolation method for specific biomarkers, as well as the need to determine the most appropriate markers. Consequently, the current priority in this field is to standardize liquid biopsy-based techniques. This review will help to improve understanding of the biology of PCa, particularly the recently defined condition known as “oligometastatic PCa”. The presented review of the body of evidence suggests that additional research in molecular biology may help to establish novel treatments for oligometastatic PCa. In the near future, the treatment of PCa will require an interdisciplinary approach involving active cooperation among clinicians, physicians, and biologists.

## Introduction

### Prostate Cancer Is the Most Commonly Diagnosed Cancer in Men

Prostate cancer (PCa) is the main cause of cancer-related mortality in males and the diagnosis, treatment, and care of these patients places a major burden on healthcare systems globally. In countries with a large proportion of elderly men, the morbidity rate associated with PCa remains high (1). Clinically, PCa is highly heterogeneous, ranging from indolent tumors to highly aggressive disease (2). In many cases treatment—generally either radiotherapy (RT) or surgery—can be curative (3). Several key genetic and demographic factors such as age, family history, genetic susceptibility, and race are associated with a high incidence of PCa (4). The process of tumor metastasis includes the following steps: loss in cellular adhesion, increased motility, invasiveness of the primary tumor, entry into and survival in the circulation, entry into new organs, and eventual colonization of these organs (5). PCa reveals also a fair amount of genetic heterogeneity. There are several markers that helps to determine metastatic potential of PCa: Ki67 expression and PTEN loss (6).

Consequently, PCa is characterized by a specific molecular profile, including overexpression of the MUC1-C oncoprotein, which is associated with an enhanced epithelial-mesenchymal transition (EMT) process, ultimately leading to dissemination of the PCa cells from the primary tumor and metastases in epithelium-derived carcinoma (7, 8). The MYC oncogene also plays a role in the development and progression of PCa. The MYC promoter is controlled by DNA methyltransferase (DNMTs) (7, 9). Most primary prostate cancers are characterized by specific gene fusions (ERG, ETV 1/4 and FL1) or mutations (SPOP, FOXA1, and DH1). As a result, there are many different subtypes, with substantial heterogeneity (10). Goodall et al. (11) reported that 20–30% of lethal prostate cancers are characterized by deleterious aberrations in genes engaged in DNA repair mechanism-homologous recombination, including *BRCA2, ATM, BRCA1, PALB2, FANCA, CHEK2*, and *CDK12*. The available data show that homologous recombination deficiency-associated mutations correlate with a worse prognosis. These genetic aberrations are inherited in ~10–12% of men with lethal PCa (11). Furthermore, there are data suggesting that CDK12 inactivation may be associated with an altered tumor immunophenotype and have implications for curability after metastasis-directed therapy and sensitivity to immune-checkpoint blockade (12). While our understanding of PCa, which is primarily based on the tools of molecular biology—, has improved dramatically in recent years, efforts to better understand this complex disease have led to the identification of a new type of PCa–oligometastatic PCa. The term “oligometastasis” was first used in 1995 by Hellman and Weichselbaum to indicate the intermediate state of PCa in which the disease has extended beyond the prostate gland, but with only limited spread to distant organs. Oligometastatic disease should be considered an individual, heterogeneous entity with distinct metastatic phenotypes and, consequently, wide prognostic variability (13–15). In general, patients with oligometastatic disease typically present less biologically aggressive tumors whose metastatic potential is more limited and which are slow-growing. These patients are good candidates for more aggressive treatment approaches. However, the definition of oligometastatic PCa, which remains ambiguous, needs to be more precisely specified. For example, at present there is no defined cut-off point to define the presence of the “oligo” condition. Rather, oligometastasis is an intermediate state between intravascular circulating tumor cells (CTC) and disseminated metastasis. The factors that distinguish oligometastatic disease from other pathologic conditions include synchronous vs. metachronous metastases, the number and location of the lesions, the imaging method used, and the determination of the patient's castration status (i.e., castration-naïve or castration-resistant) (16). In most studies, the definition of oligometastatic PCa depends on the number of metastatic lesions (usually from 3 to 5) (17). However, some reports define oligometastatic PCa according to the lesion location (i.e., site-specific criteria) or the size of the metastases (18). Oligometastases can be observed either *de novo* (at the time of initial presentation) or as a pattern of restrained recurrence, also known as oligo-recurrence (19). An oligometastatic state can also be induced from a more extensive metastatic condition in cases in which systemic therapy effectively eliminates micrometastatic disease but not the gross disease (20). There is substantial evidence that several treatment approaches, including definitive directed treatments such as radical prostatectomy (RP), RT, and metastasis-directed surgery or ablative therapy, can cure patients with oligometastatic PCa (21, 22). These treatments may delay the initiation of androgen deprivation therapy (ADT), thus avoiding the potentially severe psychological and physical side effects associated with hormone therapy (23). Most men with metastatic hormone-sensitive PCa respond to ADT alone. Unfortunately, the cancer invariably recurs as metastatic castration-resistant PCa (mCRPC). Androgen deprivation activates both EMT and neuroendocrine transdifferentiation (NEtD). EMT leads to tumor progression by inducing the following processes: migration/invasion, tumor cell survival, cancer stem cell-like properties, and resistance to radiation and chemotherapy. Similarly, NetD is associated with visceral metastasis, aggressive disease, and resistance to therapy (24). Available data suggest that oligometastatic progression of tumor is mainly driven by epigenetic alterations. In turn, polymetastatic dissemination is characterized by overexpression of genes involved in cell division and cell cycle progression. The molecular classifiers ale needed to determine the oligometastatic PCa patients with a predominantly androgen receptor-driven disease and for whom ADT or abiraterone would constitute an effective treatment as a consequence of adjuvant approach. It could be in opposition to those who might rapidly progress to CRPC (25). Also gene expression profile significantly differ between renal cell carcinoma patients with few (≤8) and many (≥16) pulmonary metastases. It confirms the statement that oligometastasis constitutes a distinct condition with specific molecular-level signature (26).

**Figure 1:**
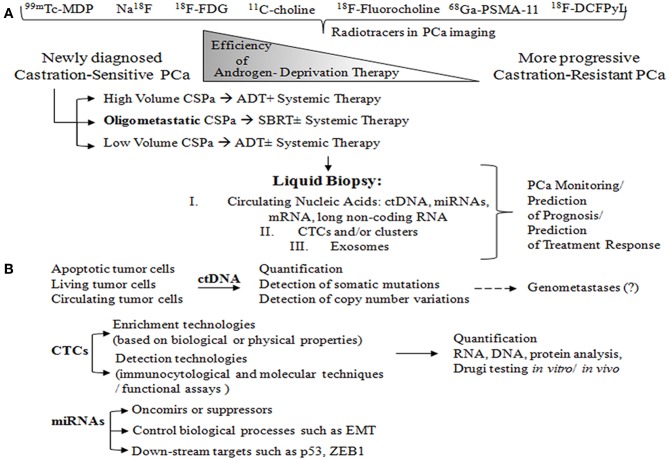
**(A)** Radiotracers commonly used for imaging in patients with oligometastatic PCa: ^99m^Tc-MDP and Na^18^F, for imaging bones with altered osteogenic activity; ^18^F-FDG, to evaluate abnormal glucose metabolism; ^11^C-choline and ^18^F-Fluorocholine, both derivatives of choline, which serve to detect metabolically active cells; ^68^Ga-PSMA-11 and ^18^F-DCFPyL, small molecule inhibitors of PSMA. PCa is characterized by the group of heterogeneous cells that are generally hormone-sensitive but can become castration-resistant. Administration of androgen-deprivation therapy (ADT) promotes the growth of castration-resistant cells, ultimately leading to the formation of castration-resistant PCa. Metastatic volume and distribution influence the treatment decision in metastatic castration-sensitive PCa (CSPC). High volume CSPC is defined as PCa with the presence of visceral metastases or ≥4 bone metastases. Oligometastatic PCa is characterized by 3–5 metastatic lesions. Liquid biopsy is a highly promising, non-invasive approach to monitoring PCa, and can be used for prognosis purposes and to predict treatment response. **(B)** Circulating tumor cells (ctDNA) arise from different sources: apoptotic cells, living cells, and circulating tumor cells (CTCs). ctDNA can undergo several different analyses including quantification, detection of somatic mutations through sequencing and digital PCR, and detection of copy number variations (array CGH). ctDNA is likely involved in transforming normal cells into tumor cells, thus leading to distant metastases (genometastases hypothesis). CTC liquid biopsy reflects *anoikis* resistance, epithelial mesenchymal transition (EMT), genomic heterogeneity, phenotypic diversity, homing and metastasis-initiating potential, invasion, and/or intravasation ability. CTC enrichment technologies are based on biological properties such as the expression of positive (EPCAM, N-cadherin, and plastin-3) and negative protein markers (CD45) as well as on physical properties (size, density, deformability and electric charges), which can be assessed through membrane and filtration-based systems, microchips, centrifugation on a Ficoll density gradient, dielectrophoresis, and spiral CTC chips. Then, CTC detection can be performed using a wide range of technologies: immunocytological (membrane and/or intra-cytoplasmic anti-epithelial, anti-mesenchymal, anti-tissue-specific marker, or anti-tumor-associated antibodies), molecular (RNA-based) (liquid bead array multi-parameter RT-quantitative PCR (RT-qPCR), and functional assays (*in vitro* cell culture- fluoro-EPISPOT technology and xenotransplantation models). The CTCs may then be subjected to a range of different analyses, including quantification, RNA and DNA-based tests, drug testing *in vitro* (organoids, 3D cultures), and drug testing *in vivo* (patient-derived xenografts). Interest in microRNAs, which can be either oncomirs or suppressors, is strong due to the potentially large impact on key biological processes such as EMT, proliferation, and cell cycle. MicroRNAs also involve downstream targets such as p53, ZEB1, EGFR, KRAS, and p73.

For these reasons, there is need to identify biomarkers that could potentially improve the treatment of advanced PCa, which often involves difficult to detect bone metastases (Figure 1A) (27). Ideally, these biomarkers should be easily obtainable in a non-invasive manner, such as those that can be collected from body fluids (e.g., serum or urine).

Circulating nucleic acids, both RNA and DNA, are extracellular nucleic acids found in cell-free serum, plasma, and other body fluids from healthy subjects or patients (28). Several genomic alterations in particular represent attractive targets associated with resistance and/or sensitivity to specific PCa treatments, including (i) phosphate and tensin homolog (PTEN) loss resulting in PI3K/AKT activation;(ii) MYC amplification correlated with metastatic phases of the disease, and finally with poor prognosis; (iii) AR mutations related to resistance hormonotherapy; (iv) TMPRSS2-ERG gene fusion; and (v) a deficiency in DNA repair genes responsible for more aggressive PCa features and worse survival (29).

Understanding the biology of oligometastases should lead to a detailed acknowledgment of molecular events responsible for the shift from an organ-confined to a disseminated metastatic disease, with future clinical implications and the possibility to identify specific biomarkers able to predict cancer evolution in patients diagnosed with a localized disease. Although specific data supporting this assumption are still lacking, different strategies are likely to be adopted to approach the molecular genetics of the oligometastatic status (30).

In this context, the primary objective of the present study is to discuss the most characteristic features of liquid biopsy as a potential tool for the diagnosis and treatment of oligometastatic PCa.

## Overview of PCa Imaging Methods

Currently, the diagnosis of PCa is based on ^99m^Technetium-methylene diphosphonate (^99^mTc-MDP) bone scan, computed tomography (CT), or magnetic resonance imaging (MRI) (31). However, these widely-used conventional imaging modalities may underestimate the burden of disease due to their limited sensitivity to detect small volume lesions (32). For this reason, a wide range of other imaging modalities may be applied to complete MRI-based diagnosis, including the following: ^11^C-choline positron-emission tomography (PET)/CT; PET/MRI; ^18^fluorodihydrotestosterone PET; ^68^Ga-labeled prostate-specific membrane antigen (PSMA); combined ultra-small superparamagnetic particles of iron oxide-enhanced and diffusion-weighted MRI (USPIO-enhanced MRI); and ferumoxytol-enhanced MRI (33).

Intensity-modulated radiotherapy (IMRT) and volumetric-modulated arc therapy (VMAT) can be used to optimize the dose distribution to more precisely target the tumor while reducing the dose to surrounding healthy tissue. The combination of high-dose RT and systemic treatment reduces the number of PCa cells in the prostate and pelvic lymph nodes (34). Active lesions can be precisely detected with MRI, ^18^F-sodium fluoride (Na^18^F), ^18^F-fluoromethylcholine (^18^F-FCH), and ^68^gallium- and prostate-specific membrane antigen-labeled PET/CT (^68^Ga-PSMA-PET-CT) (35–37). PET/CT tracers for PCa such as ^11^C-acetate, ^11^C-choline and ^18^F-choline were developed more than a decade ago; nevertheless, these tracers are still being studied to verify their reliability as imaging tools to determine disease extension in patients who develop early biochemical failure (38–40). In the present paper, we discuss the most common and/or most appealing imaging methods.

Three different techniques—^99m^Tc-MDP bone scan, CT, and MRI—are commonly used to determine the extent of disease spread in men with a high-risk primary, recurrent, or metastatic PCa. However, as mentioned above, the main limitation of these conventional techniques is their low sensitivity to detect small volume metastatic lesions (41). Additionally, these techniques are unable to detect most nodal metastases due to the aforementioned size-related limitations. ^99m^Tc-MDP is widely used to detect bone metastases. However, sodium fluoride-18 (NaF) PET combined with CT (NaF-PET/CT) has a higher sensitivity and specificity (42, 43). NaF-PET/CT ensures quick, bone-specific, blood clearance and efficient visualization of the axial skeleton. However, one important drawback of this technique is that this tracer requires an additional contrast-enhanced axial imaging to monitor soft tissue metastases (44, 45).

The glucose analog 2-deoxy-2-[^18^F]fluoro-D-glucose (^18^F-FDG), a metabolically active radiotracer, is used to image different tumor subtypes. However, its role in metastatic PCa is limited due to the low glycolytic activity of hormone-naive PCa cells, which restrains ^18^F-FDG uptake (46, 47).

The predictive value of PET/CT with choline or acetate tracers is superior to that of CT and bone scans. All PCa cells are capable of accumulating precursors of lipid metabolism and radiolabeled choline derivatives (e.g., ^11^C-choline and ^18^F-fluorocholine), which is these tracers are promising tools for the diagnosis of oligometastatic PCa (48). Although choline-based PET is characterized by a relatively low sensitivity in patients with prostate-specific antigen (PSA) values <1 ng/ml, ^11^C-choline-PET is useful in evaluating disease recurrence, with detection rates ranging from 21 to 82%, particularly when PSA values are >2 ng/ml. Nonetheless, radiolabeled choline is not completely cancer-specific due to uptake in areas with benign inflammation (49, 50). Although choline tracers provide good detection rates in PCa, particularly for local recurrences, they seem to be less specific for nodal and bone metastases because of potential uptake artifacts caused by inflammatory reactions and degenerative bone disease (51, 52). In a prospective study, Pasqualetii et al. (53) demonstrated the value of ^18^F-choline PET/CT-guided stereotactic body radiotherapy (SBRT) to treat patients with oligometastatic PCa (≤3 synchronous active lesions). Those authors found that the use of repeated salvage ^18^F-choline PET/CT-guided SBRT was well-tolerated and could delay the implementation of systemic therapy in selected patients with oligometastatic PCa (53).

Of the numerous imaging tools currently available, PSMA-targeted imaging is considered among the most valuable. This imaging modality relies on radiotracers targeting cell surface proteins, which provides outstanding sensitivity for detecting small volume PCa lesions, even in cases with low PSA values, in contrast to conventional imaging. The addition of MRI offers the potential to integrate the diagnostic path in cases with a limited accumulation of PSMA tracer (16, 54). PSMA is a type II transmembrane protein that is frequently overexpressed in PCa tissues. PSMA expression correlates with tumor stage, Gleason score, and the risk of recurrence. For this reason, PSMA is a promising target for molecular imaging, which explains the strong research interest in PSMA-PET radiotracers (55). ^68^Ga-PSMA ligand PET/CT is considered a more sensitive radiotracer compared to choline-based PET/CT, with great potential to detect metastatic lesions in recurrent PCa, especially nodal metastases (56). A retrospective study in patients with locally-recurrent and oligometastatic PCa demonstrated that ^68^Ga-PSMA ligand and PET/CT-guided RT is strongly associated with slower clinical progression, thus also delaying initiation of systemic treatment. Moreover, the preliminary results in that cohort suggest that RT with ^68^Ga-PSMA ligand PET/CT provides local control, with a substantial treatment response (i.e., lower PSA levels) without clinically-relevant side effects (57). Guler et al. retrospectively investigated the efficacy and toxicity of ^68^Ga-PSMA PET-CT-guided RT for oligometastatic PCa, finding that this technique may be a promising approach to treatment of patients with biochemically-recurrent PCa (58). Shenderov et al. (59) reported a significant role for radiotracers such as [^18^F]DCFPyL in PSMA-targeted PET. Those authors described a patient with oligometastatic PCa who had concurrent metastatic small bowel carcinoid tumor. In that patient, the lack of uptake by the carcinoid tumor proved the accuracy of [^18^F]DCFPyL as a diagnostic tool for identifying metastatic PCa (59). Dietlein et al. compared the clinical utility of [^68^Ga]Ga-PSMA-HBED-CC and [^18^F]DCFPyL radiotracers in biochemically-recurrent PCa. Compared to F-18, Ga-68 demonstrated higher availability and better image resolution, leading the authors to suggest that [^18^F]DCFPyL is a highly promising alternative to [^68^Ga]Ga-PSMA-HBED-CC in PSMA-PET/CT imaging in patients with recurrent PCa. PSMA is also expressed in gastric and colorectal cancer cells. Thus, in the near future, PSMA-selective ligands could potentially be used to image other tumor types (60). In short, [^18^F]DCFPyL is a promising approach to improving diagnostic accuracy due to the higher amount of tracer activity, longer half-life, and higher image quality attributable to the lower levels of positron emission energy of the F-18 tracer. We believe that these emerging imaging techniques are likely to improve the clinical capability to monitor disease status in PCa and to treat oligometastatic lesions.

## Treatment of Metastatic PCa

Systemic therapy, which involves ADT with or without chemotherapy in combination with new anti-androgens, remains the current standard of care (SoC) for all metastatic PCa (mPCa) subtypes (61). However, it is important to distinguish between two different conditions: (1) newly-diagnosed, low-burden mPCa, which is considered “oligometastatic” disease and (2) failure after radical treatment, which is known as “oligorecurrent” disease (62).

### Treatment of Oligometastatic PCa

The SoC for newly-diagnosed, low-volume metastatic disease is hormone therapy. However, emerging data from retrospective studies (63–65) suggest that local treatment can provide a survival benefit in oligometastatic patients. These findings have given rise to randomized controlled trials such as the STAMPEDE trial (66), which was conducted to compare SoC (ADT ± docetaxel) with or without external beam radiotherapy (EBRT) to the prostate. In unselected patients, SoC plus radiotherapy improved failure-free survival (FFS) compared to SoC alone [[HR] 0.76, 95% CI, 0.68–0.84; *p* < 0.0001] but did not improve OS. However, in the subgroup of patients with low metastatic burden (according to the CHAARTED definition) (67, 68) EBRT improved 3-year OS (81 vs. 73%; HR 0.68, 95% CI, 0.52–0.90; *p* = 0.007) and FFS (0.59, 0.49–0.72; *p* < 0.0001). In another trial (HORRAD) (69) ADT + EBRT was compared to ADT alone, with some (non-significant) improvement in OS for the EBRT arm in the subgroup (probably underpowered) of patients with low metastatic volume. Despite the limitations of these studies, such as the relatively low total radiation dose in both STAMPEDE schemes [55Gy/20 fractions [fxs]/4 weeks and 36Gy/6 fxs/6 weeks], the results support the recommendation to consider radical radiotherapy to the prostate in well-selected, newly-diagnosed oligometastatic patients. Numerous similar studies are currently underway. For example, the four-arm PEACE-1 trial (NCT01957436) was designed to compare ADT+ docetaxel ± local radiotherapy ± abiraterone (70, 71). That study is important due to the known benefit of adding abiraterone to conventional ADT for hormone-sensitive stage IV patients (72).

Another important question under investigation is the optimal approach to definitive local treatment, with two ongoing trials currently underway: (1) the phase II MD Anderson Cancer Center trial (NCT01751438), with initial results expected in 2019) (73) and (2) the phase III SWOG/NCTN trial (NCT03678025) (74), which include radical prostatectomy or EBRT as potential local interventions.

The results of the trials described above suggest that an aggressive, multimodal, multitargeted approach may be curative in well-selected patients. Such an approach would involve radical treatment of the primary tumor together with metastasis-directed therapy. In this context, two trials are currently underway—the Memorial Sloan Kettering METACURE trial (NCT03436654) (75) and the Veterans Affairs trial (NCT03298087) (76)—both of which include radical prostatectomy to the primary tumor site plus metastasis-directed SBRT, ADT, and novel antiandrogens (abiraterone, apalutamide). The details for these trials are summarized in Table 1.

**Table 1:** A selection of ongoing trials investigating radical treatment of oligometastatic PCa.

**Study**	**Phase**	**Arms**	**Estimated completion date**	**Estimated enrolment**	**Primary endpoints**
NCT01957436 (PEACE 1)—UNICANCER, EORTC	Phase III	Arm A: ADT + docetaxel (6 cycles at 75 mg/m^2^/cycle, one cycle every 3 weeks) Arm B: ADT + docetaxel + abiraterone acetate (1,000 mg/d) + prednisone (5 mg bid) Arm C: Arm A + RT (74 Gy in 37 fractions) Arm D: Arm B + RT	December 2030	1,168 pts	OS, PFS
NCT01751438—MD Anderson	Phase II	Arm A: Best systemic therapy (BST) Arm B: BST + surgery or RT (RP or prostate RT)	March 2019	180 pts (actual)	PFS
NCT03678025—SWOG/NCI	Phase III	Arm A: Standard systemic treatment (SST) Arm B: SST + prostatectomy or RT (prostatectomy within 8 weeks after randomization or RT within 4 weeks of randomization.)	October 2031	1,273 pts	OS
NCT03436654 (METACURE)—Memorial Sloan Kettering	Phase II	RP followed by: Arm A: ADT + apalutamide Arm B: ADT + Apalutamide + abiraterone acetate + prednisone Note: Cohort 1—High risk M0 pts, Cohort 2—M1 oligometastatic patients, Cohort 3—Biochemical failure after RP	February 2020	76 pts	Pathologic complete response. Minimal residual disease (MRD)
NCT03298087—Veterans Affairs	Phase II	Single arm: RP [and post-operative fractionated RT for pT = 3a, pN1, or margins [+]], + metastasis directed SBRT + ADT + abiraterone acetate with prednisone + apalutamide (total: 6 months of systemic therapy)	September 2022	28 pts	% patients achieving serum PSA <0.05 ng/mL 6 months after recovery of serum testosterone
NCT03784755 (PLATON)—Canadian Cancer Trials Group	Phase III	Arm A: SST (+ ablative therapy to untreated prostate primary for patients with low-volume metastatic disease) Arm B: SST + local ablative therapy (SBRT) to all sites of disease (including untreated prostate primary)	December 2025	410 pts	FFS

*RP, radical prostatectomy; RT, radiotherapy; SBRT, stereotactic body radiotherapy; ADT, androgen deprivation therapy; PFS, progression-free survival; OS, overall survival; FFS, failure-free survival*.

### Treatment of Oligorecurrrent PCa

A range of salvage approaches can be used to manage loco-regionally recurrent PCa, including radiotherapy (particularly brachytherapy and SBRT) and prostatectomy. The treatment of choice for distant metastases is ADT due to its well-documented capacity to improve survival, despite the sometimes significant adverse effects (77). However, given the nature of low-volume oligometastatic disease, there is growing interest in escalating metastasis-directed treatment (MDT) in these patients. The European STOMP trial (78) randomly assigned hormone-naïve patients who had developed asymptomatic, recurrent PCa (1–3 extracranial metastatic lesions) following radical treatment to surveillance or MDT of all detectable (by choline-PET/CT) lesions (SBRT: 30 Gy/3fxs or surgery). The primary end-point was ADT-free survival (the indication for ADT was symptomatic progression, progression to >3 metastases, or local progression of baseline-detected metastases). The ADT-free survival was 21 months in the MDT arm vs. 13 months for the surveillance arm. The ongoing American ORIOLE trial, which has a similar study design, is expected to report results by 2020 (79).

The SABR-COMET trial (80) included patients with different types of advanced (stage IV) cancer (prostate, lung, breast, and colorectal cancer, among others) with oligometastatic disease (1–5 metastatic lesions). The patients were randomized to SoC (palliative radiotherapy to symptomatic sites ± additional chemotherapy) or stereotactic ablative radiotherapy (SABR) ± additional chemotherapy. The findings showed better overall (41 vs. 28 months) and progression-free survival (PFS; 12 vs. 6 months) in the SABR arm. These results were considered a breakthrough in the treatment of PCa.

The typical progression pattern in all of the treatment arms in the aforementioned trials was polymetastatic progression, a pattern that suggests that there was a subgroup of patients whose “oligometastatic” disease was actually only “oligo-visible” lesions (present from the start of treatment), and thus these patients should not have been enrolled in those trials because they did not actually met the enrolment criteria. This observation is relevant because the precise definition of oligometastatic disease depends on the quality of the imaging technique. Nevertheless, based on the current evidence, it seems reasonable to escalate MDT (using either SBRT or surgery) to all detectable metastatic lesions in patients with low-volume disease and good performance status.

One approach to identifying the most appropriate treatment scheme for a particular candidate could be liquid biopsy to assess certain biomarker. Despite the undeniable efficacy of the new anti-androgens, the primary resistance (81) it was already revealed that AR variants, copy number changes or mutations are linked to abiraterone and enazalutamid resistance (82–84). Genomic profiling based on cfDNA reflects patient's response to enzalutamide and disease progression. Aberrations such as AR amplification, multiple AR mutations, RB1 loss as well as AR-L7202H/AR-T878A mutations, PI3K pathway alterations, and CTNNB1 mutations are strongly associated with primary and acquired resistance, respectively (85).

In the ongoing Dutch CABA-V7 study (NCT03050866) (86), the investigators select AR splice variant 7 (AR-V7) positive patients from among a group of patients with mCRPC in whom docetaxel treatment has failed. These patients are then scheduled to receive second-line cabazitaxel treatment instead of new antiandrogens (e.g., enzalutamide, abiraterone). The hypothesis underlying this approach is that AR-V7 messenger ribonucleid acid (mRNA) expression in CTCs appears to be associated with lack of response to androgen receptor-targeted therapy (87). We explore the concept of liquid biopsy in the next section.

## Liquid Biopsy—A New Diagnostic Tool For Patients With Metastatic Disease

Generally, accurate diagnosis of oligometastatic disease constitutes a problematic procedure because all radiological modalities are strongly limited by certain thresholds for the detection of size-limited metastases. Nieder et al. (88) performed a retrospective study that involves 34 patients with a limited number (maximum five in total) of distant metastases. They proved that elevated level of LDH and reduced hemoglobin is associated with shorter survival, whereas in multivariate analysis, hemoglobin outperformed LDH. This work shows that serum biomarkers may reflect the total burden of malignant disease (88). Thus, the biomarkers that will allow to objectively and unequivocally identify oligometastatic patients are needed (89).

Based on the principle of liquid biopsy, various different analytes can be characterized in serum samples, including the following: CTCs and mature endothelial cells or tumor-educated platelets; circulating cell-free DNA (ccfDNA), such as circulating tumor DNA (ctDNA); circulating cell-free RNA (ccfRNA) or extracellular vesicles (exosomes) as well as a cargo of exosomes (nucleic acids and proteins) (90). CTCs can be analyzed at the DNA, RNA and protein levels and can also be expanded *in vitro* for drug testing or other purposes (91). For these reason, it seems likely that liquid biopsy will be increasingly used in the future as a non-invasive tool for the diagnosis, treatment, and follow-up of PCa patients (Figure 1B). The main advantages of liquid biopsy is that it is easy, low-cost, and fast; in addition, it has substantial potential to overcome tumor heterogeneity or the multifocal nature of some tumors by providing a more systemic view of the tumor burden (92). Additionally, the routine implementation of liquid biopsy in clinical practice in PCa will probably occur more rapidly than in other cancer types, mainly because the concept is not completely new in PCa, as blood-based testing (i.e., PSA) is already in routine clinical use. Aside from blood-based markers, urinary testing is also likely to become common prior to radical prostatectomy due to the ease and convenience of collecting urine samples. In this regard, the anatomical location of the prostate is highly advantageous (93, 94). Despite the many advantages of measuring PSA levels, this does not provide information about the biological features of the PCa, and it loses its predictive nature in mCRPC setting (95). For this reason, newer blood-based and urinary markers would provide valuable data.

### The Key Properties of Circulating Tumor Cells

**Figure 2:**
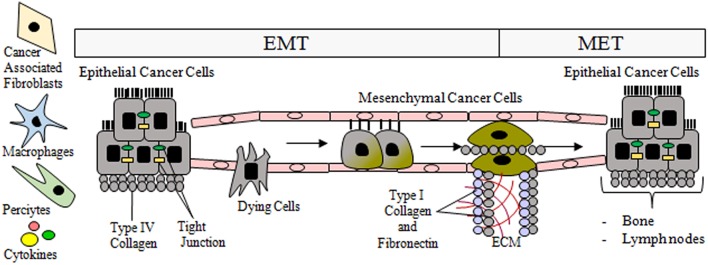
During epithelial mesenchymal-transition (EMT), tubular epithelial cells downregulate the expression of adhesion molecules (E-cadherin, claudins, and cytokeratins), and upregulate mesenchymal markers such as vimentin and fibronectin. Then they undergo a reorganization of the cytoskeleton and morphological alterations. The transition process from epithelial to mesenchymal phenotype is gradual and includes intermediate morphological changes. Post-EMT invasive cells leave the primary tumor and enter the circulatory system via trans-endothelial intravasation where the primary tumor cells can migrate to a capillary or to the lymphatic system, subsequently exiting circulation. As a result of MET, the migrated epithelial cells colonize new tissue (e.g., bones, lymph nodes) and become micrometastases that eventually develop into full tumors.

**Figure 3:**
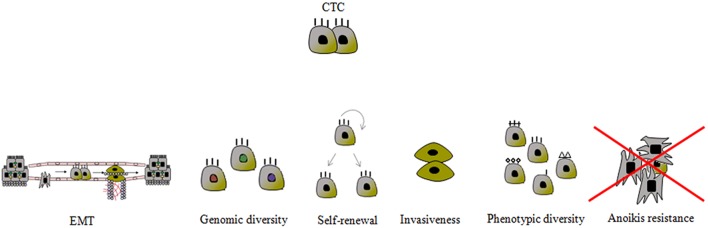
Schematic presentation of the most important CTC characteristics. The mechanisms to prevent *anoikis* are still unknown. CTC phenotypic diversity refers not only to the epithelial-mesenchymal transition (EMT) phenomenon, but also to the expression of proteins associated with apoptosis, proliferation, invasion and chemotaxis, and it is closely related to genotypic variability.

The prevalence of CTCs, which range in size from 4 to 50 μm, is from 1 to 10 CTCs per 10^6^-10^8^ white blood cells. Due to their small size and relatively low prevalence, it is challenging to detect, quantify, and particularly isolate single cells. These tasks are further complicated by the EMT process that CTCs undergo, which means that the expression profile of their markers evolves over time (Figure 2). Consequently, it is essential to identify additional mesenchymal markers of CTCs that are activated and upregulated during EMT (Figure 3) (90, 96). Current markers considered characteristic of CTCs include the following: N-cadherin (membrane protein from the cadherin family), vimentin (structural cytoskeletal protein), nuclear localization of β-catenin, and transcription factors such as SNAIL, SNAI2, TWIST, zinc finger E-box binding homeobox 1—ZEB1, ZEB2, and TCF3.

The “anoikis” process is responsible for suppressing the expansion of oncogenically transformed cells by preventing proliferation at migrating locations. However, migrating tumor cells resistant to anoikis induction survive and grow at inappropriate locations. Resistance to anoikis can be considered a hallmark of metastatic cancer cells, particularly since anchorage-independent growth of tumor cells is a classic characteristic of various human cancers, including PCa (97, 98). Recognition of critical anoikis signaling events will enable the therapeutic optimization of anoikis*-*targeting to impair PCa metastasis prior to it can start (99).

**Figure 4:**
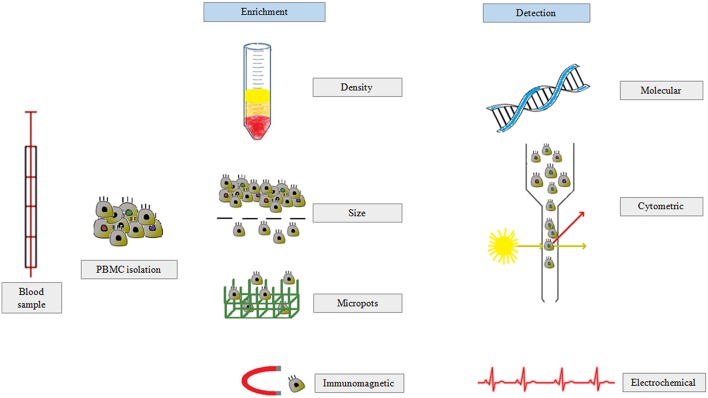
CTCs are isolated from peripheral blood in three main steps: (i) separation of peripheral blood mononuclear cells, (ii) enrichment of CTC population based on density, size, or antibody expression, and (iii) CTC detection by molecular biology, flow cytometry, or electrochemical methods.

The method most commonly used to isolate CTCs is based on the presence of the epithelial marker EpCAM (Figure 4) (100). However, this method has a serious drawback as published data suggest that there are additional CTC subpopulations or CTC-like cells that lack EpCAM expression, which means that this test might give false negative results. Notably, members of the family of cytokeratins (CK8, CK18, CK19) are also considered gold standard markers of CTCs (101).

Most CTCs are single cells. However, they can also form clusters known as microemboli, particularly in the circulating blood of patients with advanced disease. CTC clusters play an essential role in the formation of metastases (91). However, only 2.5% of CTCs are capable of forming micrometastases and just 0.01% of these lead to macroscopic metastases (102). Recent research findings have demonstrated the existence of a mechanism that includes acquisition of an “organ-mimetic phenotype” by CTCs. This phenotype involves the presence of cells in the primary tumors with different capacities to metastasize to distant organs (103). Tumor cells reduce the number of hematopoietic stem cells (HSCs) by driving their terminal differentiation. Platelets are likely to have a protective effect on CTCs. In turn, leukocytes may have either a protective or cytotoxic effect on CTCs (103, 104). Of all the targets in the field of CTC characterization in PCa, the androgen receptor (AR) has received the most attention from researchers. The transition of advanced CRPC into an aggressive neuroendocrine phenotype with low AR expression is defined as a final event in PCa progression, a phenomenon that occurs most frequently in patients with metastatic disease (91, 105).

Disseminated tumor cells (DTCs), which are CTCs that can home to the bone marrow, represent another interesting potential target. DTCs can be detected in peripheral blood years before clinically-detectable metastases are identified (106). Bone marrow is a dormancy-inducing organ, but it is also a frequent site of overt metastases in breast, prostate, and lung cancer. DTCs can be found in the bone marrow and can spread through the lymphatic system in higher numbers than CTCs. Moreover, a larger quantity of DTCs can be isolated from bone marrow than CTCs from blood (107, 108). However, since DTCs are measured at a specific time point and at a definite location, these cells are thought to be less suitable than CTCs as a prognostic factor. Additionally, blood sampling is substantially less invasive than marrow sampling, and for this reason blood tests are a more convenient and comfortable procedure for routine use in patients (109). One of the most critical roles of CTC biology is that played by chemoattraction. The crucial member associated with chemoattraction is the CXC-chemokine receptor type 4 (CXCR4)-stromal cell-derived factor 1 axis, which is engaged in homing of both CTCs and DTCs in bone marrow (110, 111). DTC markers and phenotypes are highly heterogenous: for example, in PCa, the CD45^−^EpCAM^+^ population of DTCs has an erythroid-like rather than the expected prostate gene expression profile. Moreover, two distinct populations of DTCs may occur: (1) CK^+^, which presents features of EMT such as high expression of Twist, TGF-β, Slug, and Zeb1 or (2) CK^−/low^, AR^−^, neuroendocrine, and/or CSCs phenotypes (112).

Tumor cells with phenotype characteristics involving both epithelial and mesenchymal properties present the greatest plasticity and are classified as cancer stem cells (CSCs), which consist of the subpopulation of the CTC fraction. As a consequence of the EMT process, CTCs lose EpCAM and CK markers, leading to differentiation from the CSC subgroup. CSCs are largely resistant to radio- and chemotherapy-based treatment, and thus responsible for most metastases. CSCs remain CD44^+^/CD24^−/low^–and in the case of unique tumorigenic properties—also ALDH^+^ (113–115).

An interesting trial was conducted by Mandel et al. (116). They determined the pre- and postoperative value of CTC enumeration in 33 patients with hormone naïve oligometastatic PCa (HNoMPC) undergoing cytoreductive prostatectomy (CRP). Importantly, they revealed that CTC enumeration both at diagnosis and 6 months after CRP as a prognostic value seems to have an advantage over biomarkers such as PSA, ALDH, and bone-specific alkaline phosphatase. In patients being at hormone-sensitive stages, a cut-off of two or more CTCs per 7.5 ml blood was characterized by highest explanatory power, whereas, up to five of eight CTCs seems to constitute an accurate number in the prediction of OS in mCRPC patients (116).

### The Main Methods of CTC Detection

CTC enrichment techniques are based on morphological- or immunological-based characteristics (Figure 4). Detection methods are divided into cytometric- or nucleic acid-derived approaches. Immunomagnetic isolation is correlated with the expression of selected antigens, including EpCAM^+^ or CK19 and CD45^−^. The major disadvantage of this method is that not all CTCs present the EpCAM marker on the cell membrane. Additionally, EpCAM expression may be barely detectable, and thus part of the CTC population will be lost during manipulation (117, 118). The CellSearch® system includes EpCAM-labeled iron oxide nanoparticles to enrich CTCs and to detect cells labeled with anti-CK and anti-CD45 antibodies. This system also includes an analysis based on cytomorphological features such as the size, presence of the nucleus, and appropriate nuclear to cytoplasmic ratio (119, 120). The detection of ≥5 CTCs per 7.5 ml by CellSearch® is considered a marker of metastatic PCa (121). A highly promising CTC marker not downregulated during EMT is actin bundling protein plastin 3, which is not expressed by blood cells. Isolation of CTCs is also based on depletion of normal hematopoietic cells by bead-conjugated antibodies against CD45 and CD15, which are not expressed by CTCs (122). However, none of these enrichment methods ensure that a pure population of tumor cells will be obtained. Thus, for all separation techniques, a detection method that can distinguish CTCs from other cells is essential (117).

The sensitivity of flow cytometric techniques is lower than reverse transcription-polymerase chain reaction (RT-PCR). Because PCR-based methods measure the whole amount of DNA, it is not possible to use this technique to differentiate between DNA-derived apoptotic and viable cells. However, RT-PCR enables the detection of mRNA derived only from viable cells, and thus identification of nucleic acid from cells responsible for the metastatic process. RT-PCR may be more useful to characterize CTCs rather to detect them. Consequently, a multi-marker approach is required to identify CTCs (102, 123). The expression level of full-length AR or splice variants is correlated with clinical outcomes and response. AR-V7, the most commonly expressed AR-V, is associated with resistance to abiraterone and enzalutamide and inhibition of the AE axis, and thus with poor OS (124, 125). Scher et al. (126) assessed the correlation between AR-V7 on CTCs and outcomes and survival in mCRPC patients. They evaluated the role of AR-V7 expressed and localized on CTCs as a treatment-specific marker for response and outcomes between ARS inhibitor and taxanes. mCRPC patients with AR-V7- positive CTCs demonstrated resistant prostate-specific antigen response, shorter time on therapy, shorter radiographic progression-free survival and inferior overall survival. Thus, there is a strong treatment-specific interaction between AR-V7 status and taxane administration (126). Later, those research group suggested that AR-V7 protein on CTCs can identify patients who may live longer with texane chemotherapy vs. ARS inhibitor treatment. Consequently, the AR-V7 assay can be used to select a taxane or ARS inhibitor and provide individual patient benefit (127). Although immunocytochemistry is the gold standard for the tumor diagnostics, this is a time-consuming technique. Most CTCs do not possess Ki-67, a proliferation antigen, which explains why they are resistant to chemotherapy (128).

Currently, label-free methods based on cell size and morphology, which is why as three-dimensional (3D) microfilters and bilayers seem to be desirable approaches. Their efficiency depends on pore size, rigidity, and blood flow rate. The blood flow rate may cause both “squeezing” of CTCs through pores as well as leukocyte accumulation and blood clotting if inappropriately chosen. Microfluidic devices (CTC- or herringbone-chip) isolate CTC clusters based on difference in physical features and specific antibody-binding features (91, 129). Another promising approach is represented by Obayashi et al. (130). They taking advantage of an inexpensive and highly sensitive microfluidic CTC-capture polymeric chip, which was subsequently used in CTC enumeration in 2 ml blood samples derived from 14 metastatic PCa patients. Unfortunately, there exist a notable limitation of potential using this method in blood deriving from oligometastatic PCa patients: in this case significantly fewer CTCs can be detected in comparison with multiple distant metastases PCa samples (average CTC count was 48 cells/ml) (130).

In general, morphology-based enrichment techniques are not recommended for routine clinical procedures because a relatively large amount of cells are lost during these procedures (102).

### Cell-Free DNA-Based Approach

Cell-free DNA (cfDNA), also known as cell-free-tumor DNA (ctDNA), originates from apoptotic and necrotic cells, and is considered a biomarker of advanced solid tumors. These DNA fragments may be released by apoptotic cells in the form of nucleosomes (131). Tumor development results in an elevated level of necrosis, leading to higher levels of circulating tumor DNA. The cfDNA fragments are composed of both genomic and mitochondrial DNA and these can be used to analyse microsatellite instability, loss of heterozygosity, mutations, polymorphisms, methylation, and DNA integrity. Patients with PCa present much higher levels of cfDNA than healthy individuals, which means that a relatively straightforward cfDNA-based analysis involves the simple quantification of cfDNA (132, 133).

CfDNA, which consist of ~160 base pairs in length, are routinely isolated from different body fluids, mainly blood. In most studies, cfDNA is used to detect point mutations and copy number variations, and can also detect larger chromosomal aberrations. Screening of epigenetic changes in cfDNA is a growing focus of research (90, 134). In healthy individuals, cfDNA fragments are isolated from apoptotic cells, with the release of uniformly-truncated fragments (~185–200 base pairs). By contrast, cfDNA fragments derived from malignant cells as a result of necrosis, mitotic catastrophe, autophagy, and mitochondrial catastrophe vary in size because they are randomly and incompletely digested (7, 135). The majority of studies concerning ctDNA focuses on following patients with cancer rather than on assessment of its utility in screening settings. ctDNA is elevated in >85% of patients with advanced forms of main cancer types. However, it has been also proved that a considerably smaller fraction of patients diagnosed with earlier stages of cancer have ctDNA at the detectable level in their plasma (136). Measuring the quantity of cfDNA can be challenging due to the high degree of fragmentation and low circulating concentration. Although cfDNA fragments in serum are about 2–24 times higher than in plasma, plasma is probably a better source of cfDNA, which is caused by a clotting process leading to contamination from cells in serum. Thus, plasma has lower concentrations of background wild-type DNA (137, 138). It is important to emphasize that increased concentrations of cfDNA have also been detected in physiological and, crucially, in non-cancerous pathological conditions such as heart dysfunction connected with heavy smoking or exercise (138, 139). The half-life of cfDNA is relatively short (<2 h) and thus may provide a real-time measure of tumor status. Furthermore, high cfDNA levels are correlated with shorter PFS and OS in men with mCRPC. For all these reasons, cfDNA constitutes a promising non-invasive liquid biopsy technique that could provide an accurate quantitative and qualitative tumor assessment, which in turn would permit more personalized treatments (140).

In healthy individuals, the normal cfDNA concentration level is <5 ng/ml, mainly derived from hematopoietic cells. Elevated levels of cfDNA in plasma are characteristic in patients with PCa, in whom the plasma contains both circulating tumor DNA (average 30%) and DNA from healthy tissue. In patients with advanced cancer (including but not limited to PCa), the average concentration of cfDNA is 17 ng/ml, with the highest concentration in patients with mCRPC (53 ng/ml) (141).

Living tumor cells (e.g., lymphocytes) release DNA continuously and automatically, which may explain why it is possible to detect cfDNA in early-stage cancers. In addition, the amount of cfDNA increases as the tumor grows, which also supports the hypothesis that cfDNA is derived from living tumor cells. The hypothesis that cfDNA is released from CTCs is supported by three main factors: first, both cfDNA and CTCs are characterized by identical genetic mutations; second, CTCs can escape macrophages and easily enter the bloodstream; third, blood that contains CTCs also contains cfDNA. These facts strongly suggest that CTCs may be an alternative source of cfDNA (142). In one study, plasma DNA samples were obtained from 16 CRPC patients and subjected to targeted next-generation sequencings, which identified aberrations such as mono-allelic deletions of 21q22 and *NKX3.1*, point mutations in *FOXA1, TP53*, and *SPOP*, as well as *PTEN*-deletion. That study found that those genomic lesions are involved in early carcinogenetic processes, suggesting that multiple distinct tumor clones give rise to metastatic disease (143). The released ctDNA might play the role of an intercellular messenger and could either integrate into the genome of a host cell resulting in genetic instability or it would bind to receptors leading to transformation of target recipient cells at distant locations. This effect gives the basis for the theory of “genometastasis.” Higher cfDNA levels have been observed in advanced tumor stages than in patients with non-metastatic disease. However, the increase in serum, but not in plasma, cfDNA concentration in advanced tumor stages strongly correlated with leukocyte counts (144).

### RNA—Another Interesting Area of Liquid Biopsy

As an emerging biomarker for disease biology, RNA possesses several advantages over DNA, including the following: (i) RNA expression is a dynamic process that changes according to the internal needs of cancer cells; (ii) the expression profile of specific RNA molecules or fragments such as lncRNA is highly tissue- or disease state-specific; (iii) this RNA-based approach enables investigation of non-coding RNAs, fusion transcripts, splice variants, and RNA editing events (93, 145). Currently, there is available a set of commercial tests such as Polaris®, Decipher® Prostate Cancer Classifier and OncotypeDX® Prostate Cancer Assay based on mRNA profile of tissue derived from PCa patients. The evaluation of prognostic ability in stratifying patients at risk of metastasis or biochemical recurrence after primary treatment relies on genes engaged in cell cycle progression and on genomic classifier and genomic prostate score (146).

Micro RNAs are small single-stranded non-coding RNA molecules (18–22 nucleotides in lenght) which control transduction of mRNA. Altered microRNA is related to cancer development (e.g., tumor growth, differentiation, adhesion, apoptosis, invasion) and metastasis formation. Therefore, microRNAs have a great potential to serve as biomarkers. The tumor tissue releases those molecules to the biological fluids such as blood, urine, saliva) inside exosomes. Therefore, they are ideal candidates used in non-invasive biopsies, known as liquid biopsy (89). Oligometastatic PCa is a specific biologic condition that relies on microRNA-mediated impairment of prometastatic epithelial plasticity programs, primarily EMT. MicroRNA expression has excellent potential to identify those patients most likely to remain oligometastatic after MDT (147). A mouse model of oligometastatic disease showed that miRNA-200c is responsible for the transition from the oligometastatic to polymetastatic phenotype, which is why miRNA will likely become a useful marker of poor prognosis in patients with oligometastases (148). Another study used a model of breast cancer lung colonization to investigate the role of miRNAs, finding that miR-127-5p, miR-544a, and miR-655-3p all appear to play an essential role in identifying cells with a low potential for malignancy (149). Another study found that expression levels of miR-23b, miR-449a, and miR-449b may also be strong candidates as predictors of survival after SBRT in patients diagnosed with oligometastatic cancer (150).

Cancer-associated RNAs can be detected in the peripheral blood of patients with PCa and for this reason they are good prognostic or predictive biomarkers. Endogenous small RNAs include miRNAs, transfer RNAs, ribosomal RNA, and RNA fragments (151). In patients with CRPC, miR-21, miR-221, miR-1290, and miR-375 are all upregulated (152). Research has shown that docetaxel-resistant patients with shorter survival present higher levels of the miR-200 family or decreased/unchanged post-therapeutic levels of the miR-17 family (91). CTC-related miRNA may provide relevant information about the subtype origin of these cells, which can be crucial for clinical practice (153).

A comprehensive overview of miRNAs and their role in EMT process in PCa was provided by Sekhon et al. (154). We are of the opinion that it is important to pay a particular attention on this precisely described miRNAs because in the near future they can serve as reliable biomarkers in diagnosis of progreesion of oligoPCa patients. They can be also potential targets for treatment of oligoPCa condition using gene therapy such as knock-down and overexpression tools: EMT-inhibiting miRNAs: miR-200 family, miR-205 and miR-203, let7 family; EMT- promoting miRNAs: mir-301a, miR-21, miR-32 (154).

Due to fact that miR-200 family is highly involved in EMT process. We would like to take close look to this miRNA family because its members are good candidates as biomarkers in prognosis of oligoPCa patients. The miR-200 family consists of five members: miR-200a,−200b,−200c,−141, and−429 that play a crucial role in cancer initiation and metastasis. However, it can be distinguished their dual role in tumor biology: they take part in metastatic colonization and on the other hand, in suppression of cell transformation, cancer cell proliferation, migration, invasion and tumor growth and metastasis. For this reason, they are regarded as both oncogenes and tumor suppressors and they are commonly dysregulated in human cancers. The inhibition of miR-200 induces a mesenchymal-like spindle cell morphology, accompanied by an increase in *ZEB1* expression and cell migration. Overexpression of miR-200 members represses EMT by directly targeting and downregulating *ZEB1* and *ZEB2 via* miR-200-binding sites located within their 3′ UTRs, resulting in enhanced E-cadherin expression and inhibition of tumor cell migration and cancer cell motility (155, 156).

Long non-coding RNAs are defined as >200 nucleotides RNA transcripts. These RNAs are composed of the following subtypes: antisense RNAs, pseudogenes, and long intergenic non-coding RNAs (lincRNA) (157). The association between these RNAs and cancer progression is explained by their influence on mechanisms such as chromatin remodeling, transcriptional co-activation or repression, modulation of protein activity, post-transcriptional regulation, or as decoy elements (158). LncRNAs are involved in transforming normal prostate cells into prostate intraepithelial neoplastic cells, and in the development of localized tumors and progression to advanced metastatic disease. This phenomenon is triggered by aberrant lncRNA expression, which influences the balance of protein-coding genes engaged in processes such as proliferation and apoptosis, thus facilitating cellular transformation (159, 160). This advancement in transcriptome analysis resulted in taking advantage of many lcnRNA associated with PCa. The PCA3 lncRNA was the first recommended lncRNA- based urinary biomarker (in this case: oncogene) for prostate biopsy in patients (with a serum PSA values >3 ng/ml). Apart from that, other lncRNAs have been identified as promising biomarkers in PCa development and progression: lncRNA RP11-543F8.2, PCAT1, PCGEM1, MALAT1, PCAT-18, lncRNA FR0348383, SChLAP1, lncRNA LOC400891, lnc-MX1-1, PCAT14, lincRNA-p21, CCAT2, HCG11, ATB. We pay particular attention to PCAT-18 that constitutes a potential therapeutic target and biomarker for metastatic PCa (146).

Several lncRNAs—not only prostate cancer antigen 3 (PCA3) but also prostate cancer gene expression marker 1 (PCGEM1), and prostate cancer associated ncRNA transcript 1 (PCAT1)—are highly prostate-specific and thus attractive candidates for use as biomarkers (161). Based on urine samples from patients with PCa, the non-coding RNA SChLAP1 (second chromosome locus associated with prostate-1) is highly expressed in ~25% of these cancers, and particularly common in metastatic PCa, thus suggesting that SChLAP1 plays a critical role in the development and progression of PCa (162).

Tumor suppressor growth arrest-specific 5 (GAS5)-encoded small nucleolar RNAs (snoRNAs) are upregulated during PCa progression. High *GAS5* expression levels are believed to promote basal apoptosis and enhance the response to apoptotic stimuli. By contrast, a low level of *GAS5* expression diminishes the intensity of programmed cell death in response to physical and chemical stimuli (163).

## A Brief Comment on the Abscopal Effect and Its Consequences on Metastatic Process in PCa

### Biological Background of the Abscopal Effect

Localized treatment of the primary tumor leading to a radiation-induced immunological response is responsible for regression of metastasis disease. This phenomenon, known as the abscopal effect, contributes to the induction of antitumor immunity, thereby resulting in broader systemic effects (164, 165). The abscopal effect is associated with T-cell mediated and antigen-specific immune reactions. These immune reactions are caused by RT-induced macrophages and dendritic cells during tumor necrosis. The local inflammation involves more efficient antigen cross-presentation and immune activation leading to CD8+ cytolytic T cell responses. However, the precise underlying the mechanism of the abscopal effect, which we describe in more detail below, remains unclear (166, 167).

The abscopal effect may induce a range of inflammatory cytokine cascades and immune effector cell activation induced by immunogenic cell death. Ultimately, this leads to the destruction of unirradiated tumor cells. Tumor-derived peptides stimulate the immune system by recruiting T-cell receptors. In addition, other co-stimulatory molecules, such as B7/CD28 and tumor necrosis factor (TNF) receptor family members, are involved in the propagation of additional activating signals. The release of mixed proinflammatory mediators (e.g., interferons and interleukins) plays an essential role in generating a specific immunologic response that can disrupt non-targeted tumor growth *via* an IR-mediated global stress response (168). Immunogenic cell death involves the release of high levels of antigens. This process is triggered by enhanced antigen presentation through the increased expression of MHC I on the tumor cell surface. Cytokine modulation also plays a role in enhancing the migration and function of effector CD8+ T cells. Consequently, delivering higher doses per fraction to the target lesion seems to have a curative impact on the nearest microscopic lesions. However, anti-tumor immunity can be attenuated by PD-1 blockade or deficiency, which may induce tumor-specific CD8+ T-cell immunity. These findings suggest that combining anti-PD-1 blockade and local RT can lead to the better systemic tumor control (169, 170). Nevertheless, some studies suggest that the abscopal effect is relatively rare due to the immunotolerance of the tumor, which results in a reduced systemic immune response. However, treatment with immune checkpoint inhibitors might overcome this immunosuppression, thus converting an immunologically “cold” tumor into a “hot” tumor (171, 172).

The application of immunotherapy, particularly immune checkpoint inhibitors, can enhance the systemic anti-tumor response to RT. For this reason, the combination of RT and immunotherapy is considered a highly promising approach. Patients with mCRPC benefit from the combination of anti-CTLA-4 (ipilimumab) immunotherapy with RT (single fraction 8 Gy) (173). PD-1 expression can be detected on T cells in response to signals derived from the tumor microenvironment; negative signals transmitted from PD-1 to T cells help to reduce cytotoxicity, leading to immune tolerance. Consequently, PD-1 blockade obviates T cell inhibition, thereby promoting an anti-tumor immune response (174). While CTLA-4 blockade leads to the broad systemic activation of T cells, it can also lead to strong immune cell infiltration and immunopathology in non-malignant tissue. By contrast, T-cell activation caused by PD-1 or PD-L1 seems to evoke a more subtle effect. However, studies that have evaluated the combination of RT and simultaneous blockade of the PD-1/PD-L1/CTLA4 have reported conflicting results due to the additional effect of combined immune checkpoint blockade on the abscopal tumor response (175).

Moreau et al. (176) described an interesting new approach to prime the abscopal effect. Those authors found that multifunctional smart radiotherapy biomaterials (SRBs) loaded with immunoadjuvants (anti-CD40 monoclonal antibody) increased the abscopal effects of RT in a mouse model of lung cancer. SRBs are likely to benefit many patients, especially those with metastatic disease, based on the rationale that SRBs enhance the abscopal effect by sustainably delivering an immunoadjuvant payload directly into the tumor microenvironment. This technique also minimizes possible systemic or overlapping immunoadjuvant-based toxicities (176). The RT-induced abscopal effect, reinforced by immune checkpoint inhibition, depends strongly on the cancer type and stage. For example, in advanced PCa, no comparable observations have been described to date (175, 177).

As noted above, the abscopal effect probably has a significant impact on reducing metastases in PCa. For this reason, we firmly believe that liquid biopsy is likely to be a highly valuable tool to assess the abscopal effect in oligometastatic PCa.

### Influence of Immunotherapy and Abscopal Effect on Oligometastatic Condition

The abscopal effect, which was first described in 1953, refers to the regression of metastatic lesions distant from the irradiated site (178), an effect that is particularly relevant in the treatment of oligometastatic disease. Although the phenomenon of out-of-field response to radiation has been known for decades, the underlying mechanisms have been unclear until relatively recently, with new evidence suggesting that the immune system is a major promoter of this effect (179). Large case series suggest that abscopal regressions are associated mainly with the most immune-dependent cancers such as melanoma, hepatocellular carcinoma, renal cell carcinoma, non-small cell lung cancer, and lymphomas (180). By contrast, in PCa, there is a lack of strong evidence for the abscopal effect, despite the immunogenic nature of PCa (181). The phase II IMPACT trial assessed autologous cellular immunotherapy based on Sipuleucel-T (Dendreon Corp.), showing that this therapy increased OS in patients with metastatic CRPC (182). For this reason, that treatment was the first U.S.-approved immunotherapy for mCRPC patients. Although the emergence of immune checkpoint inhibitors (ICI) gave rise to expectations for a new are of immunotherapy, results in PCa have been disappointing, with a weak response to single-agent ICIs (183, 184).

Data from clinical and preclinical studies have suggested that RT can activate the immune system, thereby promoting immune cell infiltration into the tumor (185, 186). This suggests that combining RT with immunotherapeutic agents would be a rational approach. Kwon et al. (187) evaluated patients with mCRPC who underwent targeted radiotherapy (8Gy, single-fraction) to bone metastases that had developed after docetaxel chemotherapy. Those patients were randomized to receive ipilimumab or placebo. Although there were no significant between-group differences in OS (11.2 vs. 10 months; HR 0.85, 0.72–1.00; *p* = 0.053)—which was the primary endpoint of the study—the authors did observe, in an exploratory subgroup analysis, a strong benefit in terms of OS for patients with favorable prognostic features (mainly those without visceral metastases), who may meet criteria for oligometastatic disease. Another option is to use “disseminated radiation” from radiopharmaceuticals to stimulate the immune system. Preclinical data on radium-223 suggest it has an immunomodulatory effect (188). Ongoing phase I studies are currently evaluating the combination of radium-223 and atezolizumab (189) and sipuleucel-T (190). In addition, one case report (191) has described a reversion of castration-resistance after radium-223 dichloride treatment.

Unfortunately, at present, no specific biomarkers have yet become available to guide prostate cancer treatment. The availability of specific biomarkers would be highly valuable in order to personalize treatment for each individual patient, but also to determine important questions such as the optimal timing of immunotherapy administration, switching between methods, and the possibility of withdrawing therapy. Studies evaluating the role of soluble markers in blood samples in PCa patients are currently ongoing (192).

## Conclusions

The main aim of the present review was to evaluate the utility of liquid biopsy for diagnostic purposes in PCa and for use in monitoring disease progression and treatment response, particularly in patients with oligometastatic PCa. This review should help to improve our understanding of the biology of PCa, particularly the recently defined condition known as “oligometastatic PCa.” In this paper, we have discussed the processes and mechanisms that are most likely to underlie the development of this distinct clinical entity. We have discussed several different possible biomarkers or gene therapy targets that could be used in future clinical practice. However, more research is needed to better elucidate this poorly-understood area. The present review of the body of evidence suggests that additional research in molecular biology may help to establish novel treatments for oligometastatic PCa. In the near future, the treatment of PCa will require an interdisciplinary approach involving active cooperation among clinicians, physicians, and biologists. The most promising novel areas of research pertain to CTCs, cell-free DNA, and RNA. Liquid biopsies offer a rapid, non-invasive approach whose use is expected to play an important role in routine clinical practice to benefit patients. However, more research is needed to resolve the many existing discrepancies with regard to the definition and isolation method for specific biomarkers, as well as the need to determine the most appropriate markers. Consequently, the current priority in this field is to standardize liquid biopsy-based techniques.

## Author Contributions

ES wrote the parts concerning biological aspects. MK wrote the parts concerning clinical issues. AG designed figures. WS gave a main concept, supervised work, and she is a grant recipient. All authors took part in preparation and modification of figures and manuscript.

### Conflict of Interest Statement

The authors declare that the research was conducted in the absence of any commercial or financial relationships that could be construed as a potential conflict of interest.
